# Editorial: Changes in the auditory brain following deafness, cochlear implantation, and auditory training, volume III

**DOI:** 10.3389/fnhum.2025.1715135

**Published:** 2025-10-29

**Authors:** Fawen Zhang, Qian-Jie Fu, Ji-Hye Han, Ravi Samy, Jing Xiang

**Affiliations:** ^1^Department of Communication Sciences and Disorders, University of Cincinnati, Cincinnati, OH, United States; ^2^Department of Head and Neck Surgery, University of California, Los Angeles, Los Angeles, CA, United States; ^3^Laboratory of Brain and Cognitive Sciences for Convergence Medicine, Hallym University Medical Center, Chuncheon, Republic of Korea; ^4^Division of Otolaryngology-Head and Neck Surgery, Lehigh Valley Health Network, Allentown, PA, United States; ^5^Division of Neurology, Cincinnati Children's Hospital Medical Center, Cincinnati, OH, United States

**Keywords:** electroencephalography, cochlear implant, cortical auditory evoked potential, music training, biomarker

During the life span, many factors (e.g., excessive noise exposure, ototoxic drugs, infections, aging, and genetic diseases) can lead to hearing loss (Willott et al., 2001; Morzaria et al., 2004). A cochlear implant (CI) is a prosthetic device used to treat severe-to-profound sensorineural hearing loss. A CI bypasses the damaged cochlear and directly stimulates the auditory nerve with a small number of electrodes placed in the cochlea from the basal toward the apical regions, mimicking but never fully replacing the function of thousands of inner hair cells that are sensitive to fine frequency differences of sounds. The spectrally degraded sound information from the CI is one main technological limitation that leads to poor performance on music perception and complex speech perception (Limb and Roy, 2014; Abdulbaki et al., 2023).

One major concern in CI research is that the clinical outcomes show substantial individual differences (Shafiro et al., 2025). In this Research Topic, one paper provided evidence that the cortical areas in different regions respond to CI stimulation differently, which may partially explain the variability observed in CI outcomes. Nourski et al. used intracranial electroencephalography (iEEG) in adult neurosurgical epilepsy patients who underwent chronic iEEG monitoring of potential seizure foci to investigate cortical processing of clear speech and spectrally degraded speech. The spectrally degraded stimuli, created using a noise vocoder to parcel clear speech into several frequency bands, were used to simulate CI stimuli. During the experiment, the iEEG electrodes were used to provide neural responses from different brain regions to the stimuli and the participants were asked to provide behavioral response for speech perception. The results showed a variability in performance for vocoded speech, allowing the identification of good and poor performance on CI stimuli. The iEEG data showed that poor performers had brain responses restricted to clear speech, while good performers had brain responses to all stimuli, suggesting variability in CI outcomes is in part attributed to the variability in cortical processing of CI stimuli.

While the brain can passively adapt to CI stimuli after implantation via passive exposure to sounds in the environment, active auditory training has been suggested for maximizing CI outcomes (Fu and Galvin, 2008; Bernstein et al., 2021). Auditory training with music stimuli and practice, i.e., music training, may enhance sensitivity of the auditory system to weak pitch and timbre information, acoustic cues important for speech and music perception (Abdulbaki et al., 2023). Previous research has demonstrated that short-term music training, as well as musical exposure both before and after cochlear implantation, can positively influence speech and music perception in CI users (Abdulbaki et al., 2023; Zhu et al., 2025). In this Research Topic, Gfeller and Mallalieu used an unusual patient-centered research method to develop and administer questionnaires with open-ended questions around lived experiences of pediatric CI users who acquired exceptional auditory capabilities through years of intensive music training. With the information collected from the CI users and their parents, the authors generated a model of music-based learning for pediatric CI recipients in which sustained music training affected by intrinsic (e.g., attitude toward music) and extrinsic (e.g., peers and parents) factors play important roles in the successful CI outcomes. The results indicated importance of motivational factors in the music training effects, which should be considered in the development of music training for pediatric CI users.

To objectively assess how the brain adapts to CI stimuli and auditory training, neurophysiological measures have been suggested in the literature. Electroencephalograph (EEG) is the best suitable noninvasive neurophysiological tool for CI users, as other noninvasive neuroimaging techniques are either incompatible with the CI or only indirectly measure brain activities.

Using EEG techniques, one can record the cortical auditory evoked potentials (CAEP) elicited by the stimulus onset (the onset-CAEP) or a change contained in a sound (the acoustic change complex, ACC). In this Research Topic, Wang et al. recorded the onset-CAEP and ACC in more than 100 participants including typically developing children and young adults with normal hearing, as well as older adults with normal hearing or mild-to-moderate hearing loss. The stimuli were white noise that contained a spatial (location) change in the middle. Results showed that the measures (peak latency and amplitude) of the ACC, rather than those of the onset-CAEP, were correlated to degree of the sound shift. The ACC differs among participant groups, indicating that the cortical processing of sound spatial changes is affected by aging and development factors. This paper supports earlier findings that the ACC may serve as an objective neural marker for cortical processing of auditory changes.

In CI users, both the onset-CAEP and ACC can be evoked by acoustic stimuli presented via loudspeakers or electrical stimuli directly sent to the CIs. Jeon et al. examined both responses with acoustic stimuli (speech /u/-/i/ and /i/-/u/) presented via loudspeakers to CI users and normal hearing (NH) listeners (both children and adults). Results showed similar developmental patterns for both onset-CAEP and ACC, with ACC maturing at a later age in CI users than in NH listeners. This finding suggested that CIs support typical development of cortical responses. The authors concluded that these CAEPs can be used as objective methods to monitor the development of the central auditory system. Saravanan et al. examined the onset-CAEP elicited by electrical stimuli (or electrically evoked late latency response, eLLR) delivered to the CI electrodes at different regions of the cochlea (apical, middle, and basal) in a group of children. The results showed that the CAEP was smaller for the basal stimulation compared to that for middle and apical stimulation. There was a positive correlation between speech recognition scores and the CAEP peak amplitudes for apical stimulation, suggesting that the CAEP may serve as a biomarker in evaluating auditory cortical development.

In summary, this Research Topic discussed effects of music training on auditory perception in CI users and objective measures of brain activities in individuals with or without CIs. We look forward to future editorial work that promotes the understanding of neural substrates of auditory training and if it is possible to use the neural biomarkers to monitor the effectiveness of auditory training and even to guide the design of effective auditory training programs.
